# Factors associated with cervical cancer screening among women aged 25–60 years in Lao People’s Democratic Republic

**DOI:** 10.1371/journal.pone.0266592

**Published:** 2022-04-07

**Authors:** Jom Phaiphichit, Phimpha Paboriboune, Sengchan Kunnavong, Phetsavanh Chanthavilay

**Affiliations:** 1 Lao-Oxford-Mahosot Hospital-Wellcome Trust Research Unit (LOMWRU), Microbiology Laboratory, Vientiane, Lao PDR; 2 Center Infectiology Lao Christophe-Mérieux (CILM), Ministry of Health, Kaoyod Village, Sisatanak District, Vientiane Capital, Lao PDR; 3 Lao Tropical and Public Health Institute, Kaoyod Village, Sisatanak District, Vientiane, Laos; 4 Institute of Research and Education Development, University of Health Sciences, Vientiane Capital, Lao PDR; National University of Singapore, SINGAPORE

## Abstract

**Background:**

Despite cervical cancer being a major public health concern in the Lao People’s Democratic Republic (Lao PDR), screening coverage is very low. The reasons and factors for this are unknown. This study aimed to identify factors associated with uptake of cervical cancer screening among women aged 25–60 years.

**Methods:**

The case-control study was conducted among women aged 25–60 years in Vientiane Capital and Luang Prabang province from March 15 to May 31, 2018. A total of 360 women were included in the study, a ratio of two controls per case. The cases were women who had undergone cervical cancer screening over the last five years. The controls were women who had never been screened or screened more than five years before, matched to the cases with residency and age (± five years). The cases were selected from central and provincial hospitals and the controls from the same community and districts where the cases resided. Conditional logistic regression was used to determine factors associated with cervical cancer screening.

**Results:**

The mean age was 42.37±9.4 years (range: 25–60), 66.67% were women from Vientiane Capital, and 86.11% were married. The common reasons for not being screened were the absence of clinical signs and symptoms (45.28%) followed by never having heard about cervical cancer (13.33%). In the multivariable analyses, we found that having sexually transmitted infections (AOR = 3.93; 95% CI = 1.92–8.05), receiving recommendations for screening from health workers (AOR = 3.85; 95% CI = 1.90–7.78), a high score for knowledge (AOR = 7.90; 95% CI = 2.43–25.69) and attitude towards cervical cancer prevention and treatment (AOR = 2.26; 95% CI = 1.18–7.16), and having a car to travel (AOR = 2.97; 95% CI = 1.44–6.11) had a positive impact on undergoing cervical cancer screening.

**Conclusion:**

Gynecological consultations, increased knowledge and positive attitudes result in women undergoing screening. Therefore, health education and advocacy for cervical cancer prevention should be provided to women.

## Introduction

Cervical cancer is a major public health concern due to high in morbidity and mortality, ranking as the second most common cancer among women aged 15 to 44 years [1]. Globally, approximately 2.8 billion women aged 15 years and older are at risk of developing cervical cancer [1]. It is estimated that every year 527,624 women are diagnosed with cervical cancer and 265,672 die from it [1]. In the Lao People’s Democratic Republic (PDR), cervical cancer ranks third in terms of morbidity and mortality for females aged 15 to 44 years [2]. According to the latest available data, about 320 new cervical cancer cases are diagnosed and 182 cervical cancer deaths are reported annually [2]. However, no comprehensive interventions have been implemented to address this burden, despite the existence of human papillomavirus (HPV) vaccines, screening and treatment.

Cervical cancer is caused by HPV [3]. More than 90% of people with HPV infection contract the virus through sexual contact [3]. Abnormal cervix cells develop even in the absence of signs and symptoms, occurring only at a late stage, making it challenging to treat [3]. However, the disease is curable if detected early [4, 5].

Though approximately 84% of Lao girls aged 10–14 years old have been vaccinated for HPV [6], cervical cancer screening is still necessary due to the incomplete protection offered by HPV vaccines. HPV vaccination only protects 70% of women from cervical cancer [7]. Screening techniques include: Visual Inspection with Acetic acid (VIA), Pap smear and HPV testing for high-risk HPV types. Among these methods, VIA and rapid HPV DNA testing are favored in developing countries due to their cost and ease of manufacture [8]. Due to the non-availability of comprehensive treatments for cervical cancer in Lao PDR, early diagnosis through screening would reduce related morbidity and mortality [9]. Studies show that screening women between the ages of 30 and 49 years, even just once, can reduce deaths from cervical cancer [9]. Scaling-up twice-lifetime screening and cervical cancer treatment would reduce deaths by 34.2%, averting 300,000 deaths by 2030 in low and middle-income countries (Ref). However, sustaining screening programs and maintaining the appropriate coverage is challenging in these countries [9].

The latest estimate by World Health Organization (WHO) indicated a very low cervical cancer screening uptake in Lao PDR. It was estimated that only 3.2% of 25–64 year-old women were screened every three years [2]. The non-availability of a national cancer management program may partly explain this issue. Precancerous screening through Pap smear is available in central hospitals, and VIA in some provincial hospitals (Luang Prabang, Champasak and Vientiane Province), where training for medical doctors was previously provided by a gynecologist [10]. However, the factors and reasons for the low rate of cervical cancer screening are unknown in the Lao context. Therefore, this study identifies factors associated with cervical cancer screening among women in Lao PDR.

## Materials and methods

### Study design and population

A matched case-control study was conducted among women aged 25–60 years living in Vientiane Capital and Luang Prabang province, Lao PDR, from March 15 to May 31 2018. Cases were had been screened for cervical cancer at the gynecology outpatient department (OPD) of Setthathirath Hospital and Luang Prabang Provincial Hospital. The controls were women who had never been screened or had been screened more than 5 years previously. Cases and controls were matched by residence (district) and age (± 5 years). Two controls per case were recruited.

### Sample size

The sample size was calculated at 360 (120 cases and 240 controls) using this formula:

$$n=\left(\frac{r+1}{r}\right)\frac{p(1-p)(Z_{\beta }+Z_{\alpha /2}{)}^{2}}{\left(p_{1}-p_{2}\right)}$$

Where: Zβ refers to the power value of 80%, = 0.84; Zα/2 = 1.96 for 95% confident interval; r refers to the number of cases per control, 1 case for 2 controls; p1 is the proportion exposed in the case, = P2*OR/(1+P2*(OR-1)); p2 is the proportion exposed in the control, = 30.53%; P = (P1+P2)/2, average proportion of exposure in the population; OR is odd ratio, 2.17; and no response rate equal to 20%.

## Sampling frame

We used purposive sampling to select cases. Any case who had come to the gynecology OPDs for cervical cancer screening, either by VIA or Pap smear, was selected. Six to ten cases per day were recruited, and recruitment ended when we had the expected number by hospital: 80 from Setthathirath and 40 from Luang Prabang. The number of women screened was higher in Setthathirath than in Luang Prabang because of the higher number of women undergoing cervical cancer screening. Controls matching cases were sought from the community after finishing data collection for cases.

### Data collection process

We began our data collection by informing the relevant local authority about the objectives of our study. We worked with doctors and nurses in the gynecology OPDs every day until we had enough cases. Following data collection for cases, we interviewed eligible controls purposively selected from the communities. The heads of selected villages from the same district as the cases then informed the selected participants about our study. Local healthcare workers or village health volunteers facilitated participant selection and interviews. Interviews were conducted by the first author, a master’s student. After obtaining written consent, the interview was conducted using a structured questionnaire.

### Variable and measurements

The questionnaire was used to collect information on knowledge, attitude and perceptions about the risk and prevention of cervical cancer. The questionnaire consisted of 8 sections: socio-demographic characteristics; reproductive health; cultural-family practices; knowledge, attitude and perceptions towards cervical cancer screening; accessibility of healthcare services; and health insurance; and the reasons for not being screened among controls. The pre-test was done for about 10 interviewees at the hospital and in the community of Vientiane Capital. Inappropriate or ambiguous questions were adjusted and modified.

The knowledge score was assessed by assigning one point for each correct answer of ten questions, and was then classified into two levels, good or poor for a total score of ≥5 or <5, respectively. The attitude score was based on their beliefs and feelings towards cervical cancer, its screening practice, prevention and treatment. This was measured using the Likert scale system in which scores range from 1 for strongly disagree to 5 for strongly agree. The responses were summed up, and a total score was obtained for each respondent. The mean score was calculated and those who scored equal to and greater than the mean, or less than the mean, were categorized as having a positive or negative attitude, respectively.

### Data analyses

Data entry and analyses used Commcare and Stata software Version 14. Descriptive analyses reported the frequencies with percentages and means with standard deviation (SD) of categorical and continuous variables, respectively. Bivariate analyses were performed to identify potential independent variables for initial models of multivariate analysis of cervical cancer screening, using backward stepwise conditional logistic regression. Crude Odds Ratios (OR) with 95% Confident Interval (CI) and *p*-value were selected for multivariable regression analyses. Only variables with the p-value<0.2 were selected for multivariate analyses.

Multiple conditional logistic regression was used to determine factors associated with cervical cancer screening, and multi-collinearity was checked. The final model retained only variables with p-value less than 0.05. The results were reported in adjusted OR (AOR) with 95% CI and *p*-value.

### Ethical considerations

The study was approved by the National Ethics Committee for Health Research in the Lao PDR (Submission ID: 2018.41.MP). The purpose of the study was explained to participants who were asked to sign a consent form about voluntary participation in the research project. It was made clear that they could leave the study at any time without penalty if they were not comfortable, and their healthcare provision would not be affected in the future.

## Results

### Socio-demographic characteristics of participants

There were 360 women recruited from two provinces: 240 (66.67%) from Vientiane Capital and 120 (33.33%) from Luang Prabang. The mean age was 42.37years (SD ±9.4, range, 25–60). Occupations varied, but three areas dominated: market vendor (32.22%), housewife (31.67%) and civil staff (30.83%). Education levels were approximately equal at all levels. The majority of women were married (86.11%), Lao Loum (94.17%), and Buddhist (97.67%) with at least one child (93.33%). The average monthly family income was 2–3 million kip.

In the control group, reasons for not being screened included the absence of signs and symptoms (45.28%), being very busy (4.44%), never having heard of cervical cancer (13.33%), and having no money (1.94%).

### Bivariate analyses

Table 1 shows the results of bivariate analyses of association between participants’ characteristics and cervical cancer screening. The characteristics significantly associated with undergoing cervical cancer screening included being students/government staff/private staff, and high education levels (college or university).

**Table 1:** Bivariate analyses of association between the presence of cervical cancer screening and participants’ characteristics.

Socio-demography	Cases (%) N = 120	Crude OR	95% CI	p-value
Occupation				
Housewife/farmer/seller	70 (27.56)	1.00		
Student/ **civil staff/private staff/business**	50 (47.17)	2.48	1.50–4.08	< 0.001
Education				
Illiterate/Primary school	19 (21.35)	1.00		
Secondary school/High school	49 (28.49)	1.72	0.88–3.37	0.109
College or university	52 (52.53)	4.56	2.25–9.26	<0.001
Marital status				
Single	2 (14.29)	1.00		
Married	108 (34.84)	3.76	0.76–28.59	0.102
Divorce or widowed	10 (27.78)	2.52	0.46–13.85	0.285
Had an abortion				
No	50 (27.62)	1.00		
Y**es**	70 (39.11)	1.74	1.10–2.75	0.018
Age at first sexual intercourse				
12–17 years	10 (22.22)	1.00		
≥ 18 years	110 (34.92)	2.05	0.94–4.44	0.069
H**istory of** STIs				
No	29 (17.26)	1.00		
Yes	91 (47.40)	5.52	3.05–9.97	<0.001
T**reated for** STIs				
No	40 (19.05)	1.00		
Yes	80 (53.33)	4.85	2.91–8.07	<0.001
T**ested for HIV**				
No	64 (26.33)	1.00		
Yes	56 (48.28)	3.29	1.91–5.66	<0.001
Were **comfortable talking to a doctor**				
No	8 (10.13)	1.00		
Yes	112 (39.86)	16.56	5.05–54.48	<0.001

STIs: Sexually Transmitted Infections.

HIV: Human Immunodeficiency Virus.

Moreover, women with cervical cancer screening history were more likely to have had an abortion, first sexual intercourse at the age of 18 years and older, a history of STIs symptoms or any other gynecological illness, a treatment history for STIs or other gynecological illnesses, a history of being tested for HIV, and being comfortable talking to a doctor about cervical cancer (Table 1).

Table 2 shows that women who had heard of cervical cancer, had received recommendations for screening from a health worker, knew a screening place, had a family member with cervical cancer, knew someone diagnosed with cervical cancer, knew someone screened for cervical cancer, knew someone who had died from cervical cancer, had a knowledge score ≥ 5 and an attitude score ≥ 47 had a higher probability of undergoing cervical cancer screening compared to those without these conditions (p<0.001).

**Table 2:** Bivariate analyses of an association between cervical cancer screening and participants’ perception, knowledge and attitude.

Variables	Cases (%) N = 120	Crude OR	95% CI	p-value
H**eard of cervical cancer**				
No	5 (8.93)	1.00		
Yes	115 (37.83)	5.84	2.27–14.97	<0.001
H**eard of cervical cancer screening**				
No	10 (8.26)	1.00		
Yes	110 (46.03)	8.95	4.26–18.80	<0.001
R**eceived a recommendation for cervical cancer screening from health worker**				
No	21 (12.80)	1.00		
Yes	99 (50.51)	6.98	3.83–12.7	<0.001
Kn**ew a place for cervical cancer screening**				
No	9 (6.87)	1.00		
Yes	111 (48.47)	11.37	5.43–23.84	<0.001
Ha**d a family history of cervical cancer**				
No	99 (30.37)	1.00		
Yes	21 (61.76)	3.57	1.71–7.46	<0.001
Kn**ew someone who had undergone cervical cancer screening**				
No	53 (27.04)	1.00		
Yes	67 (51.54)	3.96	2.35–6.67	<0.001
Kn**ew someone diagnosed with cervical cancer**				
No	60 (25.21)	1.00		
Yes	60 (49.18)	3.05	1.86–4.99	<0.001
Kn**ew someone who had died from cervical cancer**				
No	61 (24.30)	1.00		
Yes	59 (54.13)	3.93	2.35–6.60	<0.001
Good k**nowledge** (**Score ≥5**)				
No	75 (25.17)	1.00		
Yes	45 (72.58)	17.98	6.42–50.47	<0.001
Good attitude (Score ≥47)				
No	35 (21.08)	1.00		
Yes	85 (43.81)	3.02	1.85–4.93	<0.001

The women who sought care at the central hospital had a higher probability of undergoing cervical cancer screening compared to those who sought care at other places (p<0.001). Women who sought care at a hospital more often (≥3 times/year) had a higher probability of undergoing screening compared to those who sought care less often (p<0.001). Women who spent less than 30 minutes traveling to the nearest health facilities had a higher probability of undergoing cervical cancer screening compared to those who spent 1–4 hours in travel time. Women who traveled to hospital by car had a higher probability of undergoing cervical cancer screening compared to women who traveled by motorbike or other modes of transport (p<0.001). Women who had health insurance had a higher probability of undergoing cervical cancer screening compared to those not insured. (p<0.008) (Table 3).

**Table 3:** Bivariate analyses of association between cervical cancer screening and participants’ accessibility to healthcare services.

Variables	Cases (%) N = 120	Crude OR	95% CI	p-value
Visited a central hospital when sick				
No	40 (26.14)	1.00		
Yes	80 (38.65)	6.35	2.38–16.92	<0.001
Frequency of seeking care at hospital				
**1–2 times/year**	66 (25.58)	1.00		
**≥ 3 times/year**	54 (52.94)	3.64	2.14–6.21	<0.001
Travel time to the nearest health facilities				
** <30 minutes**	96 (31.48)	1.00		
** 1–4 hours**	24 (43.64)	2.07	1.02–4.22	0.043
Transportation				
**motorbike**	44 (34.58)	1.00		
**car**	70 (46.36)	3.05	1.82–5.09	<0.001
**On foot/bicycle/others**	6 (20.00)	0.63	0.23–1.72	0.369
Had health insurance				
No	44 (26.19)	1.00		
Yes	76 (39.58)	1.85	1.17–2.91	0.008

### Multiple regression analyses

Table 4 presents results of the final multiple logistic regression model of factors associated with cervical cancer screening. The results of multivariate analyses showed that five variables were independently associated with undergoing cervical cancer screening. These included 1) having a history of STIs symptoms, 2) receiving a recommendation from a health worker for cervical cancer screening, 3) having good knowledge of cervical cancer, 4) having a good attitude, and 5) having a car to travel to the health facility (Table 4).

**Table 4:** Multivariate analyses of factors associated with cervical cancer screening.

Variables	Adjusted OR	95% CI	p-value
Symptoms of STIs			
No	1.00		
Yes	3.93	1.92–8.05	<0.001
H**ad recommendation from health worker**			
No	1.00		
Yes	3.85	1.90–7.78	<0.001
Score knowledge			
Low <5	1.00		
High ≥ 5	7.90	2.43–25.69	0.001
Attitude			
Poor < 47	1.00		
Good ≥ 47	7.90	1.48–4.30	0.013
Transportation			
Motorbike	1.00		
Car	2.26	1.44–6.11	0.003

STIs: Sexually Transmitted Infections.

## Discussion

Our findings demonstrated 5 factors associated with undergoing cervical cancer screening. These included: 1) having STIs symptoms, 2) had received a recommendation from a health worker, 3) knowledge and 4) attitude toward cervical cancer, and 5) travel by car to the health facility.

Adequate knowledge of cervical cancer is very important if women are to be aware of the risks, measures for prevention and the necessity of screening, particularly in those aged 35 and older. In our study, women’s knowledge was significantly associated with cervical cancer screening, similar to previous research from Canada [11, 12]. Women with better knowledge of cervical cancer are more likely to undergo screening than those without such knowledge. Providing the health education about cervical cancer prevention to women such as at the hospitals might increase their knowledge and awareness of the diseases, subsequently increasing the accessibility to the services.

Most Lao people do not go to a hospital unless they are sick or have abnormal symptoms. More than fifty percent of participants in this study had had STIs symptoms. These women were likely to undergo cervical cancer screening, similar to findings from other countries such as Spain [13], Ethiopia [14], Thailand [15], and Uganda [12]. Having STIs symptoms is an important factor in reproductive health.

Recommendations from health workers were very important as most people depend on a specialist for information about diseases. In this study, more than eighty percent underwent screening following recommendations from health workers. This is similar to results from Thailand [15], Canada [11] and Uganda [12].

Women’s attitudes and beliefs towards cervical cancer and the importance of screening could also reflect their positive attitude to receiving cervical cancer screening. Our study found that positive attitudes were significantly associated with cervical cancer screening, similar to previous studies in Thailand [15] and USA [16].

Transportation was another important factor in attending cervical screening. Modes of transport were a significant consideration. Women with cars were more likely to undergo cervical cancer screening than women with motorbikes. Hence, it can be assumed that screening is also dependent on economic factors. This also links to lack of money as a reason for not undergoing screening.

The level of education and occupation were not significantly associated with cervical cancer screening in multivariate analyses of this study. This is different from previous research from Korea [17], in which factors associated with participation in cervical cancer screening among young Korean women showed that older age, higher education level and unemployed women were significantly associated with cervical cancer screening [17], similar to a study in Taiwan [18]. These variables were removed due to strong correlation with knowledge and attitudes in the multivariate analysis model. From bivariate analyses results, occupation and education level are also important factors for undergoing screening for cervical cancer. Government staff were more likely to undergo cervical cancer screening than those from other occupations, possibly because they had more opportunity to receive information. Similarly, women who had completed college or university were likely to undergo screening for cervical cancer, as were most married women who had given birth. This was similar to previous research from Thailand [19].

In the cultural-family context, encouragement from family members, embarrassment about getting a Pap smear done, and being comfortable talking with a doctor about cervical cancer were factors in attending screening. The results of multivariate analysis show that cultural-family practices did not have a significant effect on cervical cancer screening. This is different from previous research in the USA (study of Korean-American women), which found that culture was significantly associated with cervical cancer screening [20]. This could be explained by the fact that Korean-American women have different cultural practices from Lao women. A Korean husband does not agree that his wife show her sexual organs to another person unless there are symptoms of disease. This might be found in ethnic minorities in Lao PDR, but there was very few of them participating in our study.

Our findings show that participants did not have screening for cervical cancer for the following reasons: absence of symptoms, being busy, lack of awareness of cervical cancer risk, no money, or just did not know about cervical cancer. These results are similar to previous research from Thailand [15, 21] and HIV centers in Lao PDR [22]. This finding can be explained by the participants’ poor knowledge of cervical cancer, poor attitude towards prevention of cervical cancer and poor socio-economic level. These reasons are common, not only for undergoing cervical cancer screening, but also for other health conditions as research on hypertension in Lao PDR has shown, in which patients with poor knowledge and lack of symptoms were unlikely to go to a hospital for health checkups [23]. Another study reported that the cost of healthcare service was a barrier to seeking care in Lao PDR [24]. A lack of time was another common reason in other low-income countries for not being screened [25]. Once the lack of time for screening was known, some researchers used publicity campaigns in social marketing to encourage women to undergo screening. Wichachai et al. demonstrated that awareness of cervical cancer improved in Thailand by applying the Social Marketing Theory and the Health Belief Model [26].

### Study limitations

The results of this study should be interpreted in the light of certain limitations. The first limitation was the fact that some potential determinants of cervical cancer screening could not be included, such as satisfaction with health services, trust in healthcare and influence of the media. Selecting the controls and cases in different settings might have resulted in the study population being less representative of the population. Those who sought care at the local health facilities might have personal reasons or higher levels of satisfaction than others, resulting in a greater positive effects of recommendations by healthcare workers on screening. Thus, our data could have missed important determinants which if taken into account might have reduced bias. Another limitation is that our data collection took place in only two provinces. The sample is therefore not representative of the whole country and the results cannot be easily extrapolated to other provinces.

## Conclusions

In conclusion, good knowledge and attitudes, recommendations from healthcare workers and transport type to a healthcare center are important factors associated with cervical cancer screening in women aged 25–60 years. Healthcare providers have an important role in promoting awareness of cervical cancer and encouraging women to attend screening.
